# Whole-Genome Resequencing Analysis Reveals the Local Ancestry and Selection of Kongshan Cattle

**DOI:** 10.3390/biology14121778

**Published:** 2025-12-12

**Authors:** Mengmeng Bai, Kai Yang, Xiaohui Ma, Chenqi Bian, Wei Wang, Jun Yi, Ningbo Chen, Chuzhao Lei, Xiaoting Xia

**Affiliations:** 1 Key Laboratory of Animal Genetics, Breeding and Reproduction of Shaanxi Province, College of Animal Science and Technology, Northwest A&F University, Yangling 712100, China; baimengmeng29@nwafu.edu.cn (M.B.); chenqibian1226@163.com (C.B.); ningboch@126.com (N.C.); 2 Yunnan Academy of Grassland and Animal Science, Kunming 650500, China; yk123456ai@163.com; 3 Haixi Prefecture Agricultural and Animal Husbandry Technology Extension Service Center, Delingha 817099, China; maxiaohui152@163.com; 4 Sichuan Animal Science Academy, Chengdu 610066, China; wws20062127@163.com (W.W.); yj372197981@163.com (J.Y.)

**Keywords:** Kongshan cattle, whole-genome sequencing, genomic diversity, adaptability, ancestry, candidate genes

## Abstract

Kongshan cattle is an indigenous breed from Sichuan Province, China, characterized by their excellent meat quality, high fertility, strong disease resistance, and remarkable environmental adaptability. However, their genomic diversity has not been systematically studied. In this work, we performed whole-genome sequencing of 30 Kongshan cattle from a breeding farm and integrated these data with 113 representative commercial and indigenous cattle breeds worldwide to investigate their population structure and genetic diversity. We further analyzed the ancestral contributions to the development of the breed. The population structure revealed that Kongshan cattle possess four types of ancestral components: East Asian indicine (0.5974), East Asian taurine (0.3464), European taurine (0.0483), and Indian indicine (0.0079). The population also exhibits high nucleotide diversity, second only to pure East Asian indicine cattle. We inferred the ancestry of each variable site in the genome and, in combination with integrated haplotype score analysis, identified candidate genes related to meat quality (*ME1*, *ENPP2*, *GPD2*, *PDZRN4*, and *TMTC2*), immunity (*MCM6*, *MAP3K6*, *PIP4K2A*, *CDC6*, *CDC25B*, *PTAFR*, *ZC3H10*, and *NEK6*), and environmental adaptability (*KCNJ15*, *BECN1*, *AOC2*, *DUSP5*, and *ST3GAL4*). These findings provide valuable insights into the evolutionary history and ancestral origins of Kongshan cattle and contribute to the broader understanding, conservation, and sustainable utilization of indigenous Chinese cattle genetic resources.

## Simple Summary

Kongshan cattle is a famous local cattle breed in China, renowned for its excellent meat quality and production performance. This study employs whole-genome resequencing to systematically analyze its pedigree and genetic diversity, identifying a series of selection-related genes associated with traits such as immunity, meat quality, and reproduction. The findings provide strong scientific evidence for the conservation of this breed’s genetic resources and the development of new beef cattle breeds, with significant practical implications.

## 1. Introduction

The domestication of cattle has been a key factor in the development of human civilization, laying a solid foundation for the growth and prosperity of human society [1]. Modern domestic cattle are classified into two subspecies, *Bos taurus* (taurine cattle) and *B. indicus* (indicine cattle), which are mainly distinguished by the presence or absence of a dorsal hump [2]. Archaeological and genetic evidence have shown that the Near East was a major center for the domestication of taurine cattle, whereas the Indus River Basin was the primary domestication center for indicine cattle [3,4]. Subsequent human migrations and trade activities facilitated the worldwide dissemination of these subspecies. Over the course of long-term natural selection and artificial breeding, taurine cattle diversified into European taurine, Eurasian taurine, and East Asian taurine [5], whereas indicine cattle gave rise to South Asian indicine and East Asian indicine [6,7].

Chinese indigenous cattle represent an important genetic resource, characterized by remarkable phenotypic variation and adaptation to diverse ecological environments [8]. Previous studies have analyzed the genomic variation characteristics of Chinese local cattle breeds in their native environments via whole-genome sequencing [9]. Recent research has identified strong selection signatures in genes associated with adaptation to specific environmental pressures. For instance, genes related to high-altitude adaptation, including *PDE4D* and *PPP1R12*, have been identified in Qaidam cattle [10]. In Fuzhou cattle, *PLIN5* and *PLB1* are linked to cold tolerance and energy metabolism [11]. Yunnan cattle have acquired adaptive genes—such as *HSPBAP1*, involved in survival under hot and humid conditions—through introgression with wild bovine species, particularly the Yunnan gayal [12]. In Guyuan cattle, *RBM39* has been associated with muscle development, while *NEK6* contributes to disease resistance [13]. These patterns of genetic variation provide crucial insights into the evolutionary adaptation of Chinese indigenous cattle breeds and lay a solid foundation for the conservation and sustainable utilization of their genetic resources.

Kongshan cattle constitute an important local cattle breed in Bazhong City, Sichuan Province, China, with its main production area in the karst landform region at the border of Sichuan and Shaanxi Province. Currently, the number of purebred Kongshan cattle in stock exceeds 10,000. Through long-term natural and artificial selection, Kongshan cattle have adapted well to the local environment, boasting characteristics such as good reproductive performance, strong disease resistance, excellent grazing ability, and delicious meat quality [14]. However, research on the origin, genetic background, genomic diversity, and economic traits and adaptability of Kongshan cattle is still relatively scarce. Livestock workers urgently need to conduct in-depth studies on the genetic diversity of Kongshan cattle and implement effective conservation measures.

Therefore, this study selected 30 Kongshan cattle and compared them with 113 individuals from globally representative cattle breeds, aiming to characterize the genomic features of Kongshan cattle and provide scientific insights for their conservation and genetic improvement.

## 2. Materials and Methods

### 2.1. Sample Collection and Sequencing

**Figure 1:**
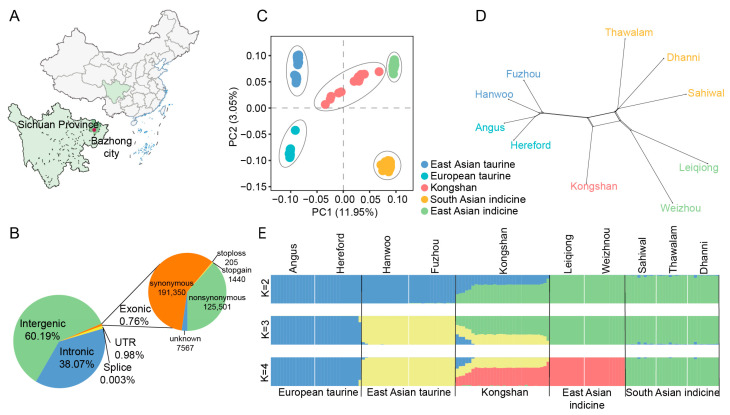
Population structure and relationships of Kongshan cattle in comparison to several possible ancestral breeds. (**A**) Geographical distribution of Kongshan cattle. (**B**) Functional classification of the detected SNPs in Kongshan cattle. (**C**) Principal component analysis between Kongshan cattle and possible ancestors. (**D**) NeighborNet graph of 10 cattle breeds using SplitsTree4. (**E**) Model-based clustering of cattle breeds using ADMIXTURE with K = 2, K = 3 and K = 4.

The Kongshan cattle are native to Tongjiang County, Bazhong City, Sichuan Province, China (Figure 1A). We selected a Kongshan cattle conservation farm in Tongjiang County for sample collection, where standardized breeding practices are followed, ensuring the preservation of the breed’s integrity. To ensure the representativeness of the samples, we referred to previous studies on livestock genetic diversity, considering individual resources, regional conditions, and ethical approval. A total of 30 adult Kongshan cattle (Table S1) were randomly selected for immobilization. Sterile sampling was performed using ear forceps, and approximately 2–3 cm^3^ of tissue was collected from the lower right section of the left ear. The tissue was then preserved in 75% alcohol and frozen in liquid nitrogen. Genomic DNA was extracted using the standard phenol-chloroform method. After quality assessment to ensure the DNA’s purity and concentration met the required standards, the samples were sent to Novogene Bioinformatics Institute in Beijing, China, for whole-genome sequencing on the Illumina NovaSeq 6000 platform. Paired-end libraries with an average insert size of 500 bp were constructed and sequencing was performed in a 2 × 150 bp format, generating reads with an average length of 150 bp.

For comparative analysis, we also retrieved publicly available whole-genome sequencing data of 113 global cattle breeds from NCBI’s (National Center for Biotechnology Information) Short Read Archive (SRA) to construct a diverse dataset. These included European taurine breeds (Angus, *n* = 14; Hereford, *n* = 15), East Asian taurine breeds (Hanwoo, *n* = 15; Fuzhou, *n* = 15), South Asian indicine breeds (Sahiwal, *n* = 10; Thawalam, *n* = 10; Dhanni, *n* = 10), and East Asian indicine breeds (Leiqiong, *n* = 11; Weizhou, *n* = 13). Additional detailed information is provided in Supplementary Table S2. In total, the dataset comprised 143 cattle genomes, which were then assembled for further analyses.

### 2.2. Mapping and SNP Calling

Initially, the raw sequencing data from 143 cattle were processed using Trimmomatic (v0.38) software [15], where low-quality reads and adaptor sequences were trimmed in paired-end (PE) mode. The specific parameters applied during this step were: LEADING:20, TRAILING:20, SLIDINGWINDOW:3:15, AVGQUAL:20, MINLEN:35, and TOPHRED33. Following quality control, the cleaned reads were aligned to the *B. taurus* reference genome assembly (ARS-UCD1.2) using BWA-MEM (v0.7.13-r1126) [16,17] with default alignment settings. The resulting BAM file was then sorted using the SortSam tool from Picard software (v2.25.5) (http://broadinstitute.github.io/picard, accessed on 1 December 2023), and duplicate reads were removed with the MarkDuplicates function, generating the final BAM file. To detect SNPs, we employed the HaplotypeCaller, GenotypeGVCFs, and SelectVariants modules from the Genome Analysis Toolkit (GATK-3.8) [18]. High-quality SNPs were then filtered using the VariantFiltration module in GATK with the following criteria: “QD < 2.0||FS > 60.0||MQ < 40.0||MQRankSum < −12.5||ReadPosRankSum < −8.0||SOR > 3.0”. Variants were further excluded if their mean sequencing depth across individuals was outside the range of < 1/3× or > 3×. Finally, the functional effects of the remaining high-quality SNPs were annotated using ANNOVAR (http://www.openbioinformatics.org/annovar/, accessed on 1 December 2023) [19].

### 2.3. Population Structure Analysis

We converted the VCF files of cattle into PLINK format using VCFtools (v0.1.12) [20]. SNPs with a minor allele frequency less than 0.01 were excluded, and linkage disequilibrium (LD) pruning was then conducted in PLINK (v1.9) (https://www.cog-genomics.org/plink/, accessed on 1 December 2023) [21] using the parameters “--indep-pairwise 50 5 0.2”. This step ensured that only independent SNPs were retained for subsequent analyses, including principal component analysis (PCA) and ADMIXTURE analysis. After pruning, the filtered SNPs were subsequently analyzed by PCA using the smartPCA program [22] from the EIGENSOFT package (v5.0) [23]. We then conducted ADMIXTURE analysis (v1.3.0) [24] on the dataset of 143 cattle, testing K values ranging from 2 to 5 to identify the optimal number of ancestral populations. For phylogenetic analysis, we calculated the genetic distances between individuals using the “--distance-matrix” function in PLINK. These pairwise genetic distances were used to construct an unrooted neighbor-joining (NJ) tree with MEGA (v11.0) [25], which was visualized using iTOL (https://itol.embl.de/, accessed on 10 March 2025) [26]. Finally, to further investigate potential population differentiation and migration events, we used SplitsTree4 software to construct a NeighborNet network based on the genetic distances between cattle breeds [27,28]. This is crucial for understanding the complex dynamics of population history.

### 2.4. Genetic Diversity Analysis

We assessed nucleotide diversity for each breed using VCFtools, applying a sliding window of 50 kb with a step size of 20 kb [29]. LD decay was analyzed by calculating the squared correlation coefficient (r^2^) for various populations using PopLDdecay (v3.42) [4,30], with default settings. Runs of homozygosity (ROH) were detected and quantified for each individual using PLINK [31,32], applying the following parameters: --homozyg-window-snp 50 --homozyg-snp 100 --homozyg-kb 100 --homozyg-density 50 --homozyg-gap 100 --homozyg-window-missing 2 --homozyg-window-threshold 0.05 --homozyg-window-het 1. The average observed (Ho) and expected (He) heterozygosity differences were calculated using VCFtools, and the inbreeding coefficient (F) for each breed was derived using the formula F = 1 − ΔHo/He. All data visualizations were generated in RStudio (v25.5.0).

### 2.5. Local Ancestry Analysis of Kongshan Cattle

We used LOTER (v1.0) [33] to determine the ancestral origins of chromosomal segments in Kongshan cattle. Based on the population structure analysis, we selected East Asian taurine and East Asian indicine—the two populations with the highest ancestral proportions—as reference populations. Using haplotype data, we calculated the length and frequency of ancestral segments from each reference population. Ancestry-specific haplotypes of individual segments were compared against the total pool of ancestry-specific haplotypes across all segments. High-frequency ancestral segments were identified by filtering for those with a significance threshold of *p* value < 0.05 and a minimum length of 1000 bp. Chromosome maps were generated using the RIdeogram (v0.2.2) package [34] to visualize segments with excessive East Asian taurine and East Asian indicine ancestry, based on the *B. taurus* genome. The filtered segments were annotated, and functional enrichment analysis of the gene list in the detected excessive segments was performed using KOBAS (v3.0) (http://kobas.cbi.pku.edu.cn, accessed on 10 March 2025) [35] to explore the functional roles and related signaling pathways of the identified candidate genes. Results with a corrected *p* value < 0.05 were considered significantly enriched.

### 2.6. Signal Detection

We used the integrated haplotype score (iHS) [36] to detect genomic regions affected by positive selection in Kongshan cattle. The iHS was computed using selscan (v2.0.3) [37] with 50 kb windows, and regions with an empirical *p* value < 0.05 were considered candidate selection regions.

An overlap analysis between genes in Kongshan cattle and those in LOTER-identified segments of excessive East Asian taurine and East Asian indicine ancestry confirmed the impact of ancestral origins on the genetic characteristics of Kongshan cattle. Tajima’s D was calculated with VCFtools for each candidate gene to further refine candidate region identification. Kyoto Encyclopedia of Genes and Genomes (KEGG) pathways and Gene Ontology (GO) terms were analyzed using KOBAS (v3.0) (http://kobas.cbi.pku.edu.cn/) to better understand gene functions.

## 3. Results

### 3.1. Sequencing, Mapping and Identification of SNPs

A total of 143 samples were used for analysis in this study, with an average sequencing depth of 12.1× and an average mapping rate of 99.37% (Tables S1 and S2).

A total of 42,800,190 biallelic SNPs were identified in the 30 Kongshan cattle samples. The functional annotation of the polymorphic sites revealed that the majority of SNPs were located in intergenic regions (60.19%) or intronic regions (38.07%). Exons accounted for 0.76% of the total SNPs, comprising 191,350 synonymous and 125,501 nonsynonymous SNPs. Additionally, 421,205 SNPs (0.98%) were detected in untranslated regions (UTRs), and 1160 SNPs were identified in splice sites (Figure 1B). Overall, Kongshan cattle presented the greatest number of SNPs, followed by East Asian indicine cattle, with 37,972,679 SNPs, whereas European taurine cattle presented the fewest, with 11,695,308 SNPs (Table S3).

### 3.2. Population Structure and Differentiation

To characterize the structure of the Kongshan cattle population, we performed PCA of the 143 animals. PC1 explained 11.95% of the total variation and was driven by differentiation between *B. taurus* and *B. indicus*. PC2 explained 3.05% of the total variation and separated the different indicine (East Asian indicine, South Asian indicine) and taurine groups (European taurine, East Asian taurine) (Figure 1C). Kongshan cattle were placed between the East Asian taurine and East Asian indicine populations.

Using SplitsTree4, a phylogenetic network was constructed based on genetic distance. The analysis results indicate that a higher proportion of indicine ancestry in Kongshan cattle brings them genetically closer to the East Asian indicine breeds (Leiqiong cattle, Weizhou cattle) (Figure 1D). The NJ tree yielded results consistent with these conclusions (Figure S1).

In the ADMIXTURE analysis, when the K = 2, the cattle populations were divided into two lineages: taurine and indicine ancestry (Figure 1E). The lowest cross-validation (CV) error occurred at K = 4, where each breed was distinctly separated, suggesting this as the optimal clustering solution. Kongshan cattle displayed clear signs of admixture, with major ancestral contributions from East Asian indicine (59.74%) and East Asian taurine (34.64%). These findings highlight the substantial influence of East Asian indicine on the genetic composition of modern Kongshan cattle and indicate a certain degree of genetic contribution from taurine cattle.

### 3.3. Population Genetic Diversity Analysis

**Figure 2:**
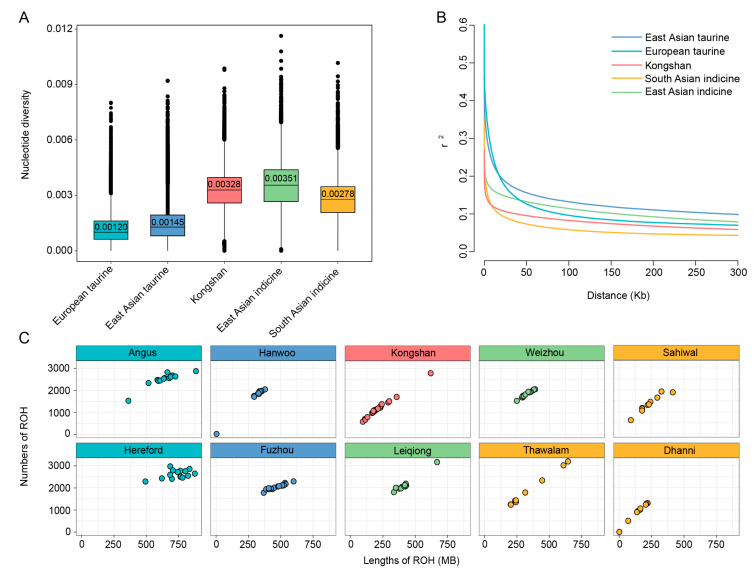
Characterization of genomes among Kongshan cattle and reference populations. (**A**) Box plots of nucleotide diversity in 50-kb sliding windows with 20-kb steps. (**B**) Decay of linkage disequilibrium on cattle autosomes estimated from Kongshan and reference populations. (**C**) Runs of homozygosity distribution in and among groups.

Nucleotide diversity analysis (Figure 2A) revealed that East Asian indicine exhibited the highest nucleotide diversity (0.00351), followed closely by Kongshan cattle (0.00328) and South Asian indicine (0.00278). By contrast, taurine cattle—encompassing both European taurine and East Asian taurine lineages—exhibited relatively lower nucleotide diversity, with values of 0.00120 and 0.00145. Regarding linkage disequilibrium (LD) patterns (Figure 2B), South Asian indicine exhibited the lowest LD levels at short distances, followed by Kongshan cattle and East Asian indicine, which showed moderate LD levels. Conversely, European taurine and East Asian taurine breeds showed higher LD levels, indicating a stronger genetic linkage within these populations.

We detected ROH to investigate the extent of inbreeding and genetic diversity characteristics of different cattle breeds. The presence of long ROHs is a result of recent inbreeding, while shorter ROHs usually reflect more ancient common ancestry [38]. The results showed notable variations in both the number and length of ROHs among breeds (Figure 2C and Table S4). In Kongshan cattle, ROHs exhibited a distinctive pattern, with the coexistence of long fragments (>500 kb) and short to medium-length fragments. This suggests that their genomes have experienced complex selection pressures and may retain genetic imprints of both ancient breeding populations and recent directional selection events.

Additionally, commercial European breeds like Hereford and Angus showed a higher number and length of ROH fragments, reflecting a higher degree of inbreeding. The estimated inbreeding coefficients (Figure S2) were consistent with the ROH findings.

### 3.4. Local Ancestry Inference of Kongshan Cattle

**Figure 3:**
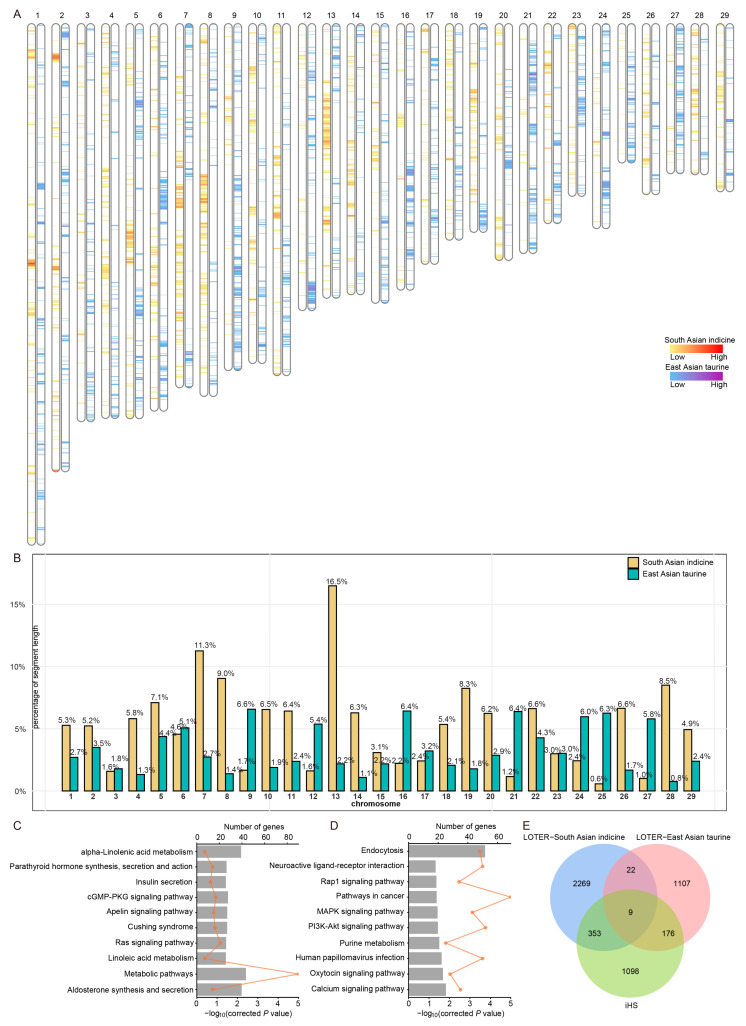
Identification of genomic segments in Kongshan cattle where the proportion of a specific ancestry is significantly higher than the overall genome proportion. (**A**) Distribution of the local segments with proportions of East Asian indicine and East Asian taurine ancestries. (**B**) The proportion of chromosome length occupied by segments with excessive East Asian indicine and East Asian taurine ancestry. (**C**) The KEGG pathways from the enrichment analysis of genes with excessive East Asian taurine proportions. (**D**) The KEGG pathways from the enrichment analysis of genes with excessive East Asian indicine proportions. (**E**) Overlap of iHS-identified genes with those from excessive East Asian indicine and East Asian taurine ancestries.

To explore the distribution of local ancestry in the Kongshan cattle genomes, we utilized the LOTER to assess the contributions of East Asian taurine and East Asian indicine lineages (Figure 3A). The chromosomes were partitioned into 1,430,516 segments, with 24,347 segments from East Asian taurine and 29,573 from East Asian indicine after applying filtering criteria (*p* < 0.05, length ≥ 1000 bp) (Tables S5 and S6). The longest segments identified were 62,869 bp for East Asian taurine and 116,785 bp for East Asian indicine. The distribution of these segments across chromosomes (Figure 3B and Table S5) revealed variability in both the number and length of segments from the two ancestral sources, reflecting a nonuniform contribution of ancestral lineages across the genome.

In the excessive East Asian taurine ancestry segments of Kongshan cattle, 1314 genes were annotated (Table S5). The enrichment results revealed significant associations with 31 KEGG pathway terms and 62 GO terms (corrected *p* < 0.05), including metabolic pathways, Apelin signaling pathway, cGMP-PKG signaling pathway, linoleic acid metabolism, and Cushing syndrome (Figure 3C and Table S7). For the excessive East Asian indicine ancestry segments in Kongshan cattle, 2653 genes were annotated (Table S6), showing significant enrichment in 11 KEGG pathways and 95 GO terms (corrected *p* < 0.05), including pathways in cancer, the PI3K-Akt signaling pathway, and endocytosis (Figure 3D and Table S8).

### 3.5. Genome-Wide Positive Selective Sweep Detection

To further investigate potential regions under selection, we employed the iHS method to identify candidate genomic regions. A total of 1636 genes were found to be associated with selective pressure (*p* < 0.05) (Table S9), with 353 genes traced back to East Asian indicine and 176 genes to East Asian taurine (Figure 3E and Tables S10 and S11).

**Figure 4:**
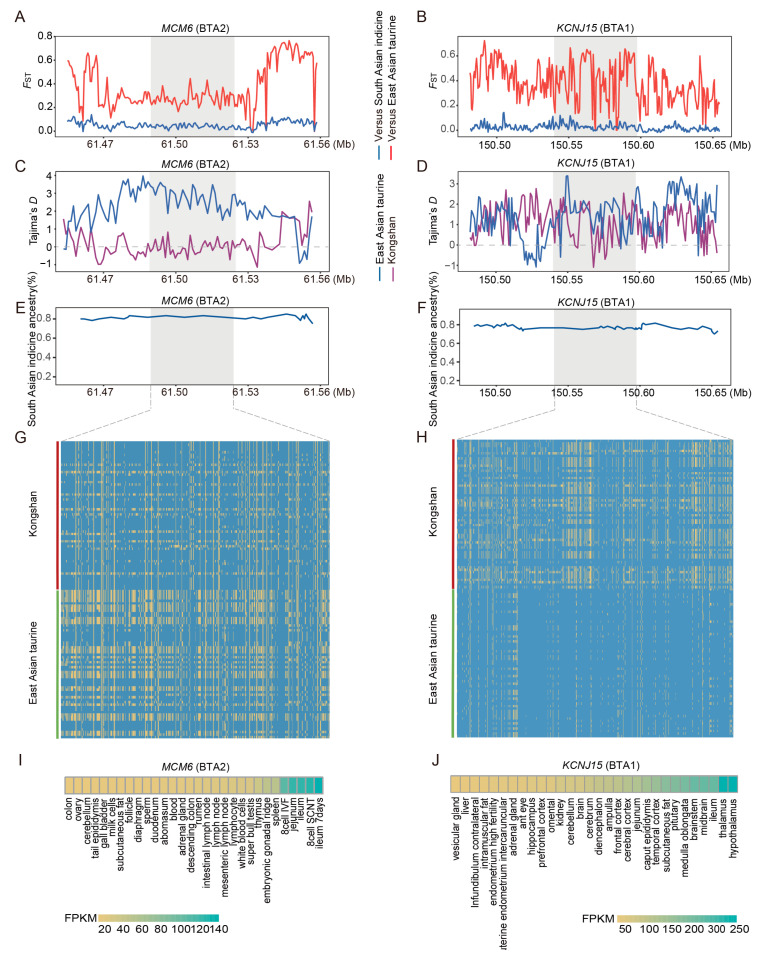
Analysis of candidate selective regions with excess indicine ancestry in Kongshan cattle. (**A**) Pairwise *F*_ST_ values around the *MCM6* gene regions. The red line indicates the pairwise *F*_ST_ values between the Kongshan and East Asian taurine cattle. The blue line indicates the pairwise *F*_ST_ values between Kongshan and East Asian indicine cattle. (**B**) Pairwise *F*_ST_ values around the *KCNJ15* gene regions. (**C**) Tajima’s D value of *MCM6* gene regions in Kongshan cattle and East Asian taurine cattle. (**D**) Tajima’s D value of *KCNJ15* gene regions. (**E**) Average East Asian indicine (%) around the *MCM6* regions. (**F**) Average East Asian indicine (%) around the *KCNJ15* regions. (**G**) Haplotype patterns heatmap of *MCM6* gene regions in Kongshan cattle and East Asian taurine cattle. (**H**) Haplotype patterns heatmap of *KCNJ15* gene regions. (**I**) Examining gene expression of *MCM6* in different cattle tissues (http://animal.omics.pro/, accessed on 10 March 2025). (**J**) Examining gene expression of *KCNJ15* in different cattle tissues.

The enrichment analysis of genes associated with an excess of East Asian indicine derived segments revealed a significant overrepresentation in several critical biological pathways, including the calcium signaling pathway, the PI3K-Akt signaling pathway, the cAMP signaling pathway, and cell adhesion molecules (CAMs) (*p* < 0.05) (Table S12). These pathways are crucial for maintaining the stability of neural and immune functions. We identified several genes related to immunity functions (*MCM6*, *MAP3K6*, *PIP4K2A*, *CDC6*, *CDC25B*, *PTAFR*, *ZC3H10*, and *NEK6*) and adaptability (*KCNJ15*, *BECN1*, *AOC2*, *DUSP5*, and *ST3GAL4*). For indicine ancestry, East Asian taurine cattle were used as the reference group for calculating *F*_ST_ values and Tajima’s D values, as well as for validating haplotype patterns. *MCM6* and *KCNJ15* in Kongshan cattle exhibited markedly high *F*_ST_ values (Figure 4A,B), low Tajima’s D values (Figure 4C,D), high indicine ancestry proportions (Figure 4E,F), and extended haplotype patterns (Figure 4G,H). Importantly, data from the Ruminant Genome Database (http://animal.omics.pro/code/index.php/RGD, accessed on 10 March 2025) [39] indicated that *MCM6* and *KCNJ15* are expressed in immune-related tissues, including the spleen and mesenteric lymph nodes (Figure 4I,J).

We performed KEGG pathway and GO enrichment analyses on 176 genes derived from excessive East Asian taurine segments. The results revealed significant enrichment of 5 KEGG pathways and 24 GO terms associated with biological processes related to adipogenesis and nervous system pathways (corrected *p* < 0.05; Table S13). Among the 176 genes, we identified key genes related to meat quality (*ME1*, *ENPP2*, *GPD2*, *PDZRN4*, *TMTC2*), neural function (*CPLX2*, *RBFOX1*, *HOMER1*), growth and development (*GLI3*, *NOP58*, *SMAD1*, *GABRG3*, *CA10*), and reproduction (*KHDRBS2*, *TGFA*, *PTPN4*).

**Figure 5:**
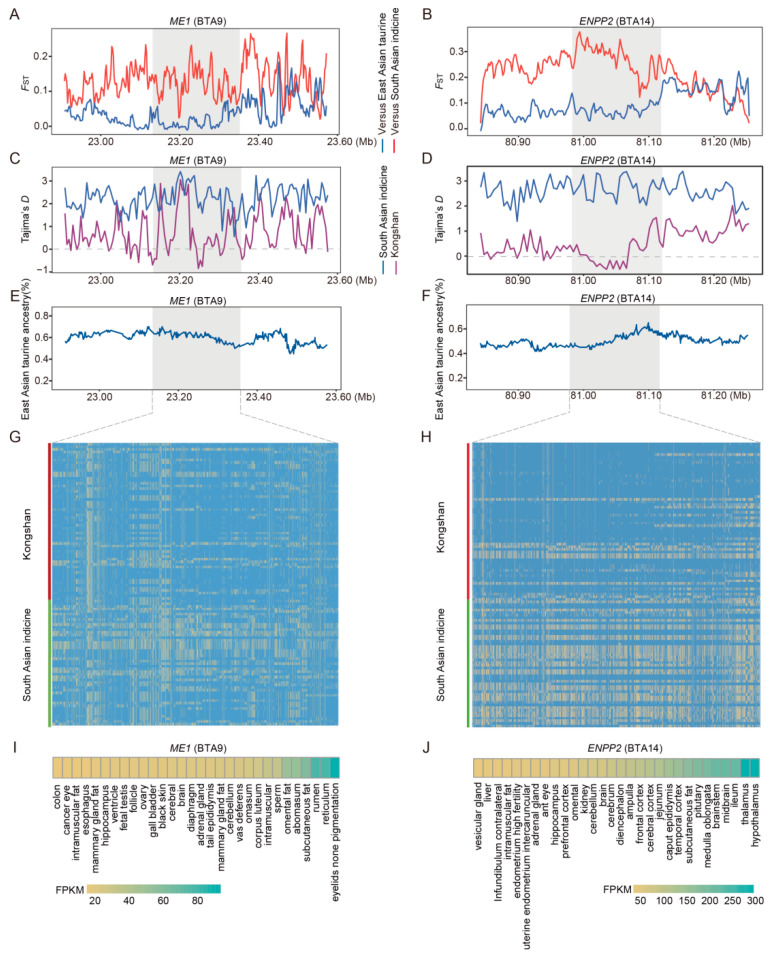
Analysis of candidate selective regions with excess taurine ancestry in Kongshan cattle. (**A**) Pairwise *F*_ST_ values around the *ME1* gene regions. The red line indicates the pairwise *F*_ST_ values between the Kongshan and East Asian indicine cattle. The blue line indicates the pairwise *F*_ST_ values between Kongshan and East Asian taurine cattle. (**B**) Pairwise *F*_ST_ values around the *ENPP2* gene regions. (**C**) Tajima’s D value of *ME1* gene regions in Kongshan cattle and East Asian indicine cattle. (**D**) Tajima’s D value of *ENPP2* gene regions. (**E**) Average East Asian taurine (%) around the *ME1* regions. (**F**) Average East Asian taurine (%) around the *ENPP2* regions. (**G**) Haplotype patterns heatmap of *ME1* gene regions in Kongshan cattle and East Asian indicine cattle. (**H**) Haplotype patterns heatmap of *ENPP2* gene regions. (**I**) Examining gene expression of *ME1* in different cattle tissues (http://animal.omics.pro/, accessed on 10 March 2025). (**J**) Examining gene expression of *ENPP2* in different cattle tissues.

For taurine ancestry, East Asian indicine cattle were used as the reference group for calculating *F*_ST_ values and Tajima’s D values, as well as for validating haplotype patterns. Among the positively selected genes, *ME1* and *ENPP2* in Kongshan cattle showed markedly high *F*_ST_ values (Figure 5A,B), low Tajima’s D values (Figure 5C,D), high indicine ancestry proportions (Figure 5E,F), and extended haplotype patterns (Figure 5G,H). Results from the Ruminant Genome Database (http://animal.omics.pro/code/index.php/RGD, accessed on 10 March 2025) indicated that *ME1* (Figure 5I) is highly expressed in tissues such as adrenal gland fat and subcutaneous fat (FPKM > 80), while *ENPP2* (Figure 5J) is highly expressed in tissues such as the hypothalamus and pituitary (FPKM > 200).

## 4. Discussion

Kongshan cattle, a local breed in China, are characterized by a strong and well-proportioned physique, strong environmental adaptability, and good production performance. They are particularly well-suited to breeding in mountainous areas and complex terrains, making them an important cattle breed in Sichuan Province. In recent years, with increased attention to the protection of local livestock and poultry genetic resources in China, studying the population structure, genetic diversity, and selection characteristics of Kongshan cattle is highly important for the conservation and rational utilization of this breed’s genetic resources.

Over the past 10,000 years, cattle domestication has been accompanied by several large-scale migrations, eventually spreading them across all continents inhabited by humans [40]. Archaeological evidence shows that taurine cattle migrated eastward from their domestication center in West Asia, reaching northern China around 5000 to 4000 years ago. About 3000 years ago, indicine cattle migrated into China, contributing to the emergence of hybrid breeds. The cattle in the Central Plains of China, such as the Qinling cattle [41], Nanyang cattle [42], Guyuan cattle [13], and Bohai Black cattle [43], are hybrid breeds resulting from the crossbreeding of *B. taurus* and *B. indicus*. This study found that the genome of Kongshan cattle is primarily composed of two ancestral types: East Asian indicine (59.74%) and East Asian taurine (34.64%). Similarly, research on Sanjiang cattle has shown that their genomic composition also exhibits significant hybrid characteristics between *B. taurus* and *B. indicus* [44]. Furthermore, the linkage disequilibrium decay results positioned Kongshan cattle between European taurine and Indian indicine, further supporting the idea that the hybridization between these two lineages is the main cause of the increased genomic diversity in Kongshan cattle, providing evidence for their complex breeding history and genomic selection pressures.

The study indicates that human activities have exacerbated the biodiversity crisis and have had a profound impact on the adaptation and evolutionary processes of species [45]. In this context, analyzing the ancestral genetic components of hybrid breeds is key to revealing adaptive evolution and phenotypic diversity [46]. In this study, we used the LOTER (v1.0) software to analyze the local ancestral genetic components of the Kongshan cattle genome, combined with selection signal analysis, to identify genetic markers associated with desirable phenotypes. The excessively retained ancestral genetic segments are associated with key biological processes such as immune response and lipid metabolism, reflecting the adaptive evolution of Kongshan cattle in complex environments. Additionally, genes related to immune regulation and environmental adaptability were identified, indicating that Kongshan cattle have gradually developed strong adaptability through natural and artificial selection. The potential of genomic selection in revealing these adaptive traits may need further updates and optimization in the future [47].

In this study, a series of genes involved in key biological processes were annotated in the excessively retained ancestral segments inherited from East Asian taurine cattle. Among the meat quality-related genes, malic enzyme 1 (*ME1*) supports lipogenesis, cholesterol synthesis, and cellular redox potential by catalyzing the decarboxylation of L-malate to pyruvate while reducing NADP to NADPH [48]. Overexpression of endogenous *ME1* has been reported to promote the biosynthesis of saturated and polyunsaturated fatty acids [49]. The ectonucleotide pyrophosphatase/phosphodiesterase 2 (*ENPP2*), an adipose-derived secretory enzyme, regulates adipose expansion, brown fat supply, and energy expenditure [50], playing a crucial role in lipid regulation [51]. Glycerol-3-phosphate dehydrogenase 2 (*GPD2*) catalyzes the conversion of dihydroxyacetone phosphate to glycerol-3-phosphate [52] and facilitates the esterification of fatty acids into triglycerides, thereby regulating triglyceride metabolism and adipogenesis [53]. *GABRG3* is associated with feed efficiency and postmortem carcass traits [54]. *PDZRN4* (PDZ domain-containing RING finger protein 4) has been identified as an important functional candidate gene for intramuscular fat content in pigs [55]. Collectively, these positively selected genes may play crucial roles in the favorable meat quality of Kongshan cattle. Interestingly, we also identified positively selected genes related to bone metabolism and development in the Kongshan cattle genome. For example, *CA10* is involved in bone mineral dissolution and resorption processes [56]. *TMTC2* (transmembrane and tetratricopeptide repeat-containing protein 2) regulates calcium homeostasis and is critical for bone and muscle development in mice [57]. These genes may contribute to the strong hooves and legs of Kongshan cattle, enabling their adaptation to mountainous terrain.

Beyond selection on meat quality traits, the environmental adaptability of Kongshan cattle has also undergone continuous selection. East Asian indicine cattle, as a primitive breed adapted to tropical and subtropical climates, carry gene loci related to heat resistance and immunity [6] and thus exhibit strong tolerance to heat and disease. These adaptations likely facilitated the introgression of indicine into taurine populations and the dispersion of indicine ancestry in crossbred animals. In this study, based on the excessively retained ancestral segments inherited from East Asian indicine, selection analysis identified a set of genes related to immunity and environmental adaptation, which may be key to Kongshan cattle survival in their local environment. Among the immunity-related genes, Minichromosome Maintenance Complex Component 6 (*MCM6*) is a DNA replication regulator that maintains cell cycle stability. Together with other proteins in the MCM family [58], it forms a hexameric complex around DNA and is implicated in cancers, autoimmune diseases, and other conditions [59]. *CDC6* and *CDC25B* are key regulators of the cell cycle. *CDC6*, along with the MCM2-7 complex, forms the prereplication complex (pre-RC) [60]. During the G1 phase, *CDC6* binds to replication origins and recruits the MCM complex to initiate DNA replication [61]. *CDC25B* promotes rapid cell proliferation by accelerating the G2/M transition and is often overexpressed in tumors [62]. *MAP3K6* (mitogen-activated protein kinase kinase kinase 6) mediates signaling pathways involved in growth, immunity, inflammation, and stress responses [63]. It activates downstream MAPK cascades in response to various stimuli, including cytokines, neurotransmitters, hormones, and microorganisms, thereby regulating multiple cellular processes [64].

Compared with ordinary cattle, Kongshan cattle not only exhibit disease resistance, immune regulation, and tolerance to region-specific pathogens but also demonstrate superior adaptability to hot and humid environments. In addition to immunity-related genes, multiple genes related to environmental adaptability that have undergone positive selection were identified, which may enhance tolerance to temperature, humidity, and low oxygen. For example, *KCNJ15* (potassium voltage-gated channel subfamily J member 15) belongs to the inwardly rectifying potassium channel family and is widely distributed in various tissues [65]. Its primary function is to maintain resting membrane potential, and it may also regulate material transport and physiological balance in the intestinal mucosa [66]. The *DUSP5* gene encodes dual-specificity phosphatase 5, a member of the dual-specificity phosphatase family, and plays a role in heat stress responses in cattle [67]. As noted earlier, these genes are involved in immune system activation and heat adaptation and are important candidates for environmental resilience. Therefore, we believe that the retained indicine ancestral fragments may help Kongshan cattle better adapt to their local environment.

## 5. Conclusions

By analyzing the whole-genome data of Kongshan cattle, we found that the breed is primarily composed of four ancestral types: East Asian indicine (59.74%), East Asian taurine (34.64%), European taurine (4.83%), and Indian indicine (0.79%). Kongshan cattle exhibit high genetic diversity and possess unique genetic resources. We also identified candidate genes related to growth, meat quality, immunity, and environmental adaptability. These findings provide a foundation for genetic breeding and resource conservation of Kongshan cattle.

## Data Availability

The raw whole-genome DNA sequencing reads newly generated in this study have been submitted to the National Genomics Data Center (NGDC), with the BioProject accession number PRJCA046865. Details of the above data and other publicly available data used in this study are listed in the Supplementary Files.

## Supplementary Materials

The following supporting information can be downloaded at: https://www.mdpi.com/article/10.3390/biology14121778/s1, Figure S1: The Neighbor-joining tree of the relationships between Kongshan cattle and possible ancestors; Figure S2: The Inbreeding coefficient for each individual; Table S1: Summary of sequencing statistics; Table S2: Summary of additional cattle sample information; Table S3: Functional annotation of the identified single-nucleotide polymorphisms (SNPs) using ANNOVAR; Table S4: Runs of homozygosity (ROHs) patterns of all individuals from each cattle geographic groups; Table S5: The gene annotation results of high-frequency segments (*p* value < 0.05, length ≥ 1000 bp) detected by LOTER software; Table S6: The gene annotation results of high-frequency segments (*p* value < 0.05, length ≥ 1000 bp) detected by LOTER software; Table S7: KEGG and GO results from the enrichment analysis of genes with excessive East Asian taurine proportions; Table S8: KEGG and GO results from the enrichment analysis of genes with excessive East Asian indicine proportions; Table S9: A summary of genes from iHS in Kongshan cattle; Table S10: A summary of genes from the overlap between iHS(Kongshan) and those from excessive East Asian indicine; Table S11: A summary of genes from the overlap between iHS (Kongshan) and those from excessive East Asian taurine; Table S12: KEGG and GO results from the enrichment analysis of genes with the overlap between iHS (Kongshan) and those from excessive East Asian indicine; Table S13: KEGG and GO results from the enrichment analysis of genes with the overlap between iHS (Kongshan) and those from excessive East Asian taurine.

## Abbreviations

The following abbreviations are used in this manuscript:

SNPs	single nucleotide polymorphisms
PE	paired-end
LD	linkage disequilibrium
PCA	principal component analysis
NJ	neighbor-joining
ROH	Runs of homozygosity
*B. taurus*	*Bos taurus*
*B. indicus*	*Bos indicus*
iHS	integrated haplotype score
KEGG	Kyoto Encyclopedia of Genes and Genomes
GO	Gene Ontology
UTRs	untranslated regions
FPKM	Fragments Per Kilobase of transcript per Million mapped reads
