# Supplementary material for: Whole-Genome Resequencing Analysis Reveals the Local Ancestry and Selection of Kongshan Cattle
Source: Biology (Basel). 2025 Dec 12;14(12):1778. doi: 10.3390/biology14121778 (PMC12730771; doi:10.3390/biology14121778)
Supplement: Supplementary file 1 [file biology-14-01778-s001.zip › Supplementary Figure.pdf]

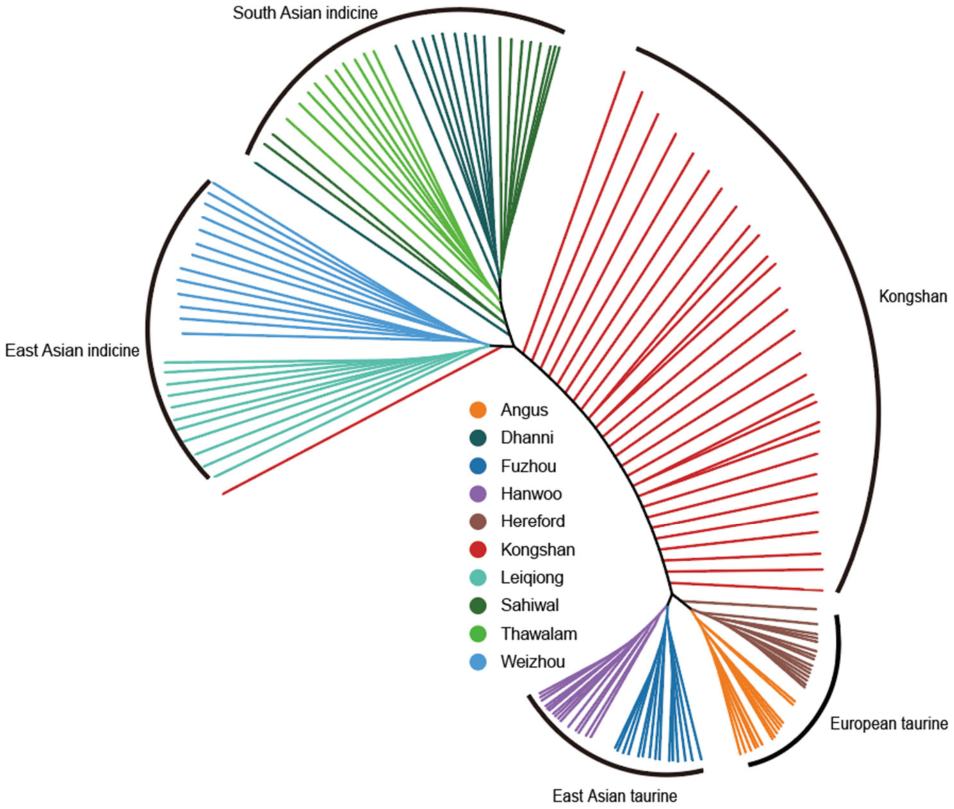

Figure S1. The Neighbor-joining tree of the relationships between Kongshan cattle and possible ancestors.

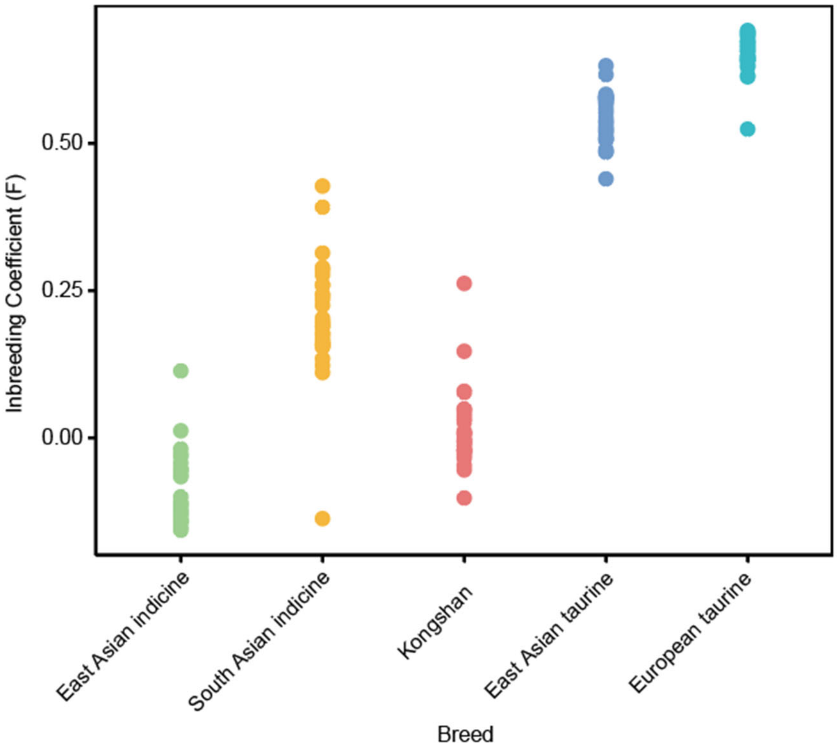

Figure S2. The Inbreeding coefficient for each individual.
